# Correlates of Falls among Community-Dwelling Elderly in Thailand

**DOI:** 10.1155/2018/8546085

**Published:** 2018-05-24

**Authors:** Titaporn Worapanwisit, Somkid Prabpai, Ed Rosenberg

**Affiliations:** ^1^Health Promotion and Health Education, Faculty of Education, Kasetsart University, Bangkok 10903, Thailand; ^2^Department of Sociology, Appalachian State University, Boone, NC 28608, USA

## Abstract

Nearly every nation is experiencing rapid population aging. One area of major concern is health; a major health risk for older adults is falling, and there are multiple negative consequences of falling. This is a global concern yet is underresearched in many nations. This study examines demographic, health, and environmental correlates of falling among community-dwelling Thai elderly. Data were collected from a sample of 406 adults aged 60–69. Significant (*p* < 0.05) fall correlates were urban residence, older age, greater BMI, impaired and uncorrected vision, chronic health conditions, medication use and medication side effects, poor muscle tone, and hazardous indoor and outdoor home environments. Results lead to recommendations for interventions to reduce fall risk that are both evidence-based and culturally acceptable.

## 1. Introduction

In the twenty-first century, most nations are experiencing rapid growth of their older populations, both in number and as a percent of the population. This growth is largely attributable to decreasing mortality due to advances in medicine and health promotion, and to fertility declines. The portion of the world's population aged 60+, 12% in 2015, is projected to rise to 22%, to 2.1 billion, by 2050.

The older adult growth rate is not uniform across nations: less-developed nations will experience more rapid growth. While those aged 60+ in developed countries grew by 29% from 2000 to 2015, less-developed countries can expect a 60–71% increase by 2030. By 2050 nearly 80% of the world's older population will live in these less-developed countries. Many Asian nations are in this latter category. By 2030, 60% of Asians will be 60+ and Asia will be home to more than half of the world's oldest-old persons (aged 80+) [1].

In this context, Thailand is typical of Asian nations. The World Health Organization [2] estimates that those aged 60+ will comprise 29.6% of the Thai population in 2050.

Population aging has serious implications for individuals, families, communities, and nations. The faster the aging, the more quickly and severely the challenges arrive, and the more difficult it is for governments to react effectively. Large-scale proactive initiatives, if and where they exist, seem the exception that proves the underpreparedness rule.

Adapting health care for an aging population is one such major challenge. As people live longer, all else equal, they will live longer with illness and other impairments. Extending healthy life expectancy is more difficult than extending life expectancy, and the “rectangularization of the survival curve” [3], where we live a long, healthy life ending in a brief, precipitous terminal drop, remains the Holy Grail of life extension.

One typology of causes of death is composed of noncommunicable diseases, communicable disease, and injuries. The World Health Organization [4] believes the distribution of noncommunicable disease deaths will remain relatively constant, while communicable disease deaths will decrease. In contrast, deaths due to injuries—the focus of this research—will grow through 2030. Injury-related deaths are a large burden on society, financially and otherwise, but many of these deaths are preventable [5]. Unintentional-injury deaths, the five leading causes of which are falls, motor vehicle traffic crashes, suffocation, poisoning, and fire [6], focus the current study further.

A fall is “an unexpected event in which the participant comes to rest on the ground, floor, or lower level” [7]. Similarly, Bekibele and Gureje [8] described a fall as an unintentional loss of balance, causing one to make unexpected or unprepared contact with the ground or floor. Weir and Culmer [9] noted that a fall results from a complex combination of individual factors operating alone or in association with precipitating environmental factors.

Falls are by far the most common cause of injury and unintentional-injury death among older persons in the United States [10], accounting for more than one-half (55%) of the total; in contrast, the second most common cause, motor vehicle accidents, accounts for only 14% [11]. Both the NCHS and the Centers for Disease Control and Prevention [12] report that the rank of falls as a cause of unintentional-injury death rises with age, from tenth among those aged 25–34 to first for those aged 60+.

Falls also have greater consequences for older adults compared to younger adults. Elderly falls may cause serious, perhaps fatal injuries such as fractures, joint dislocation, and/or head trauma. But even if falls have no lasting impact on older adults' physical health, they can have significant effects on psychosocial health, including greater fear of falling that can lead to social isolation [13]. It is difficult to determine the definitive risk factors for falling [14]. Nonetheless, fall risk factors can be trichotomized as follows: (1) intrinsic risk factors related to normal age changes, gender, race, physical problems, medical conditions, cognitive impairment, and degree of physical inactivity, (2) extrinsic risk factors, such as outdated vision correction, taking multiple prescribed medications, improper footwear, and nonuse of assistive devices, and (3) environmental risk factors, such as loose carpet, lack of grab bars in the bathroom, poor lighting, unsafe stairs, and uneven or slippery surfaces. Over one-third of elderly fall at least once annually due to various intrinsic and extrinsic risk factors [15]; 30–50% of falls among community-dwelling elderly are due to environmental factors [16].

Each year, more than one-third of the American elderly fall, with outdoor falls comprising nearly half the total—about 45%—of all elderly falls [17]. Outdoor falls comprise nearly half the total—about 48%—of all elderly falls [18]. Since Americans spend only 7% of their time outdoors [19], this is greatly disproportionate. The same is true in Thailand, where the Health Insurance System Research Office (HISRO) [20] reported that Thai women and men aged 60–69 are more likely to fall in public settings than in the home.

Most outdoor falls are precipitated by environmental factors, such as uneven surfaces and tripping or slipping on objects and usually occur on sidewalks, curbs, and streets [21]. This finding has cross-cultural support from studies in Canada [22], England [23], Norway [24], Finland [25, 26], Israel [27], and Japan [28]. Several researchers [29–31] reported that prescription medications can contribute to fall risk. Jung et al. [32] found that mobility impairments (transferring, gait imbalance, and lack of equilibrium) increase fall risk. Guzman et al. [33] related nonuse of assistive devices to increased odds of falling. In a Thai study, Kuhirunyaratn et al. [34] reported that elderly who use psychotropic medications are almost twice as likely to fall as those who do not (see also [35, 36]). Gait disturbance, visual impairment, and depression also doubled fall risk, and inappropriate indoor environment (cluttered rooms, slippery floors, nonadapted bathrooms, and dim lighting) nearly tripled the risk of falling.

Still, measures to reduce fall risk can be taken, especially at the individual and community levels. Fall-reduction improvements can be made inside and outside the home [37], often inexpensively.

Since falls incidence and prevalence rise with age, since nearly every nation is experiencing rapid population aging, and since the health, economic, and quality of life impacts of falls are significant on both micro- and macrolevels, there is a need for evidence-based research on effective fall prevention strategies and programs that reduce fall risks. Fortunately, many such studies exist, looking at the efficacy of both single- and multi-factor interventions to reduce risk factors and fall incidence. Exercise is the most common intervention used to reduce fall risk factors in community-dwelling older adults. Balance, gait, and strength training have been shown to be effective methods to reduce and prevent falls among community-dwelling older adults [38]. For example, a study of older adults [39] found that fall evaluation, balance training, home hazard management, fall prevention education, exercise, and home visitations resulted in significant fall reduction. Other multifaceted interventions, such as exercise plus dietary supplements [40] and exercise plus fall prevention education [41], yielded reduced fall rates among older adults.

Overall, falls are the leading cause of unintentional-injury deaths and can result from multiple causes, either singly or in combination. The risk of such death rises with age. Even nonfatal falls have more serious consequences for older adults than younger age groups. Major findings include (1) compared to time spent outside the home, older adults are disproportionately likely to fall there, often due to lack of familiarity and uneven surfaces; (2) prescription medications, mobility impairments, and nonuse of assistive devices are associated with greater likelihood of falling; (3) lower perceived fall risk is related to higher likelihood of falling; and (4) many evidence-based fall-reduction interventions exist. Some of these findings have been demonstrated to have cross-cultural application.

It is utopian to believe fall-related injuries can be eradicated, but one can realistically hope to reduce fall incidence and prevalence, even as nations' population age and, as they do, the risk of falls increase. The aim of this research, by reporting demographic, health, and behavioral correlates of falls for community-dwelling Thai elderly, was to add to the knowledge base of universal versus culture-specific fall-reduction knowledge, attitudes, and practices. For older people, there seem to be cross-cultural commonalities in risk of falling. But there is likely variation in preventive or intervention activities; these can depend on aspects like a society's view of aging and older people and the cultural acceptability of a proposed intervention (thus the importance of properly framing the intervention).

Two types of relationships are of interest: those that support prior research and those that vary from prior research. The latter case may merely reflect methodological variance from prior studies but also could indicate the impact of cultural variation on the risk of falls. In both cases, the findings could help identify fall prevention strategies that could reduce falls among community-dwelling older adults, promote safe, independent, healthy aging, and be culturally appropriate.

## 2. Methods

### 2.1. Sampling, Sample, and Data Collection

Thailand, a country in Southeast Asia, comprises 77 provinces and is divided into four regions: North, Central, Northeast, and South. For this study, one province—Surat Thani—was randomly chosen from the Southern region due to the residence of the lead author (“researcher”). The province has 19 districts containing a total of 131 subdistricts. Ten districts were randomly selected, and from each, a convenience sample of one subdistrict was chosen. Convenience criteria were ease of access by the researcher (transportation and relative proximity), researcher safety, and ease of access to older adults. Each subdistrict has a hospital. The researcher met with each hospital's director; with the director's consent, the researcher then met with the hospital's Health Promotion Director to obtain a list of older adults. In each subdistrict, a list of all older adults was extracted from a database made available by public health officers. Each resulting list contained 80–200 older adults; the total for all ten subdistricts was 2203.

A random sample of 50 was drawn from each list. The 500 randomly selected Thai older adults then had to meet the following inclusion criteria: aged 60–69, voluntary participation, neither a current psychiatric diagnosis nor a history of such diagnoses, and sufficiently literate to complete a questionnaire with minimal assistance. Some were excluded due to being older than 69, and some chose not to participate. Nine older adults with a current psychiatric diagnosis or history of psychiatric diagnoses were excluded from the sample. No one was excluded due to illiteracy.

After exclusions, a sample contained 406 older adults. Demographic, health, and falls history data were collected via surveys administered at participants' homes or other convenient locations (e.g., senior clubs, temples) between 15 June and 31 October 2017. Demographic variables were age, gender, marital status, residential location, education, occupational status, residence type, number of family members in household, living arrangement, income, main source of income, and health service provider. Health variables were body mass index, vision, hearing, chronic condition diagnosis, medication use, and alcohol use. Fall history variables were falls since turning 60, falls in the past six months, falls in the past three months, general cause of falls (e.g., medication side effects, hazardous home environment), specific cause of falls (e.g., footwear, drowsiness, poor lighting), general (indoors and outdoors), and specific (e.g., kitchen, sidewalk, temple) locations of falls, fall-related health problems, and treatment (if any) after falling.

### 2.2. Data Analysis

Descriptive statistics (mean, median, standard deviation, frequency, and percentage distributions) were used to describe the demographic, health, and falls history data of the sample. Data were analyzed using SPSS version 17. Due to most variables being measured at the nominal level, including the dependent variable (falls measured as yes/no or never/ever), chi-square was calculated, and *p* < 0.05 was chosen to indicate statistical significance. In some cross tabulations, a cell had a frequency of zero; these instances related never/ever fall to muscle weakness, medical condition/lack of assistive devices, hazardous home environment, footwear/clothing, and indoor/outdoor area. In these cases, the Fisher Exact Test was calculated; *p* < 0.001 was chosen to indicate statistical significance [42].

### 2.3. Research Ethics

This study was approved by the ethics committee at Boromarajonani College of Nursing, Surat Thani, Thailand. It was also approved by the Ethics Committee of the Provincial Public Health Office in Thailand for the research involving human subjects. The purpose of the research was explained to participants, as were voluntary participation, identity protection (confidentiality), and researcher contact information. Informed consent was obtained from all participants. Survey data were entered into a password-protected database on the first author's secured computer.

## 3. Results

The demographic characteristics of the sample are presented in Table 1. There were 406 community-dwelling older adults aged 60–69 (*M* = 64.60, SD = 3.20), with slightly more (52.2%) in the older category. There were slightly more females (56.0%) than males. About two-thirds (63.85%) were living in towns, the rest in cities. Nearly half (46.8%) were married, with another 31% self-identifying as part of a couple; 12.1% were single, with smaller percentages separated or divorced. Over half (53.9%) had primary school education or less. Just over one-quarter (27.9%) considered themselves working (merchant or employee), with the rest reporting themselves as unemployed, pensioners, or gardeners. Four of five (80.5%) lived in their own homes, with another 12.3% living with relatives.

The number of family members in the household where the participant lived ranged from 1 to 9 persons, with two-thirds of participants living in households with 2–5 family members. Seventy percent lived with spouses and/or children; another 22% were living with nephews or nieces. Hardly any older Thais (1.2%) lived alone. Participants' mean income was 10,953 Thai baht per month (about US$ 335). A bit less than one-third (30.7%) came from government support, followed by employment (26.6%) and children (25.4%). Thai elderly in the sample had, essentially, no savings. All but one of the 406 participants received health care from the government hospital.

Data on participants' health status are presented in Table 2. Body mass index (BMI) showed well over half (63.3%) to be overweight or obese. About three-fifths (61.3%) had normal vision. Of those with vision problems, more than four-fifths were diagnosed with presbyopia or blurry vision. Nine of ten participants had normal hearing; of those who did not, less than one in five used any type of hearing aid. The majority (60.1%) of participants had a diagnosed medical condition, with the primary ones being hypertension, hyperlipidemia, diabetes mellitus, benign prostate hypertrophy, or heart disease. Two-thirds (66.0%) used medications to help with their medical conditions, with the most common being antihypertensive drugs, antihyperlipidemia drugs, and antihyperglycemic drugs. Nine of ten participants (92.1%) reported not drinking alcohol.

Participants' fall history is described in Table 3. About three-quarters (73.9%) of the sample reported no falls since age 60, with the rest acknowledging one or more falls. Of the 26.1% reporting falls, most (21.1%) reported falling one or two times since turning 60. Only about one in ten had fallen in the six months preceding the study; even fewer had fallen in the preceding three months.

Although the great majority of the sample did not identify specific causes for their falls, some of the attributed causes, such as a hazardous home environment, were more common than others. Physical weakness, medication side effects, and lack of assistive devices were also reported. Regarding specific causes, improper footwear and “contact falls” (losing one's balance due to being jostled, bumping into furniture, etc.) were the most commonly mentioned.

More participants fell outdoors (65.1%) than indoors; about one in ten (11.3%) reported falls in both locations. As one might expect, the majority of outdoor falls (69.3%) occurred in the area surrounding the house. Indoor falls were most common in frequently used areas: kitchen, bedroom, and living room.

For Thai elderly, a fall comes with physical consequences; nearly all (97.2%) reported fall-related health problems. Of those reporting physical consequences, fortunately, most cited relatively minor problems: swelling/bruising, pain, and abrasions comprised 85.7% of reported consequences. More serious outcomes, such as lacerations, fractures, dislocations, or head injuries, were far less common, accounting for 14.3% of reported problems. Treatment behavior was quite varied: about one-quarter went to the hospital, another quarter obtained medications from a pharmacy, and another quarter used no treatment whatsoever.

Table 4 reports bivariate analyses of likelihood of falling and several independent variables. For community-dwelling Thais aged 60–69, falls are significantly more likely to occur among (1) city residents versus residents of towns; (2) older participants; (3) those with the least or the most education; (4) overweight and obese (measured by BMI) elderly compared to normal weight and underweight elderly; (5) those with visual impairment compared to those with normal vision; (6) those with a chronic health condition; (7) those using medications versus those who do not; (8) those reporting muscle weakness compared to those claiming no muscle weakness; (9) those reporting side effects from medications; (10) those with physical impairments who lack assistive devices; (11) those reporting a hazardous home environment; and (12) those using footwear or clothing that can contribute to falling.

## 4. Discussion

The sample of 406 community-dwelling Thai adults aged 60–69 had many traits that imply representativeness of the Thai population and, in fact, make this sample similar to those of other studies. There were more females than males, more in rural than urban areas, most were living in their own residences, and nearly all received health care from government facilities.

On the other hand, three-quarters of the Thai sample were married or cohabiting, educational attainment was low (over half had not attended secondary school), nearly all (about 19 of 20) lived with immediate or extended family, and over one-quarter reported their main source of income as their children (31% reported “government support”).

In addition, nearly two-thirds (63.3%) were overweight or obese based on BMI; a solid majority (61.3%) reported normal vision, and 90.4% reported normal hearing. Six in ten had a chronic condition diagnosis, and two-thirds were taking one or more medications. As might be expected in a heavily Buddhist nation, over nine in ten (92.1%) reported not drinking alcohol.

Regarding falls history, only one-quarter (26.1%) of the sample reported one or more falls since turning 60 (compared to the one-third of American elderly who fall in a given year); however, the maximum age in the sample was 69, and it can be expected that falls incidence and prevalence will rise as the sample lives into its 70s, 80s, and 90s. Despite the disproportionate likelihood of falling outside (versus inside the home) based on percent of time spent outside, the most commonly attributed cause of falls was indoors: a “hazardous home environment.”

Bivariate analyses found several factors were associated with higher fall risk: age, urban residence, low or high educational attainment, being overweight or obese, impaired vision, chronic health conditions, medication use and side effects, muscle weakness, nonuse of assistive devices, a hazardous home environment, and certain types of footwear/clothing.

One counterintuitive finding was the high percent (35.6%) of the sample with master's degrees (*n*=45) reporting ever falling (Table 4). In general, the percent reporting a fall declined as education increased. This could result from increasing educational attainment leading to less physical and physically risky jobs, better health education and health promotion opportunities, and better overall health. The literature on the education-falls relationship reaches no consensus: studies from Australia and Finland found the logically expected inverse relationship [26, 43], while other studies found a positive ([44], in America) or insignificant ([45], in Turkey) relationship between educational attainment and falls among older adults. Additional cross cultural and multivariate studies are needed to understand the relationship, if any, between educational attainment and falls among older adults.

In some ways, Thai culture can affect the risk and rate of elderly falls. Thai culture, historically and even today, is collectivistic and family oriented. In a more individualistic and independent culture, such as in the United States, the government often provides economic, health care and other supports for older adults based on the assumption that the family will not or cannot do this. Within any given nation, this shift from a more communal to a more individualistic society tends to occur with modernization [46, 47]. However, the effects of modernization on the lived experience of older adults can be and are mediated by culture.

For example, in Thailand, China, and other Asian nations including highly modernized Japan, there is a strong cultural norm of filial piety that (adult) children will honor their aging parents by caring for them, even to the point of doing as much as they can for them. Regarding the focus of this research, this operationalization of filial piety can include activities the aging parents might still be quite capable of doing themselves, leading to less physical activity, less muscle tone, and thus increased fall risk.

The data suggest recommendations to reduce fall risk for older Thais. First, the interior home environment could be modified, often at little or no cost, to reduce the risk of falling. Examples include better lighting, nonskid flooring, and grab bars/handrails in the bathrooms and on stairs. Second, the external environment could be modified, given the disproportionate number of falls that occur outside and the 70% that occur on the “house surroundings.” Smoothing/repairing uneven surfaces, and adding handrails on outside stairs or replacing stairs with ramps are examples of such modifications. Older adults could be strongly encouraged to maintain a healthy weight, to seek treatment for vision problems, to learn about and practice chronic disease self-management, and maintain regular physical activity. When it comes to clothing and footwear, safety should be a greater concern than vanity.

As is well known, the physical and psychosocial consequences of elderly falls are significant and negative, a drain on the time, energy, and resources of the older adult and his/her family. But these consequences, and thus the recommendations above, pertain to more macrolevels as well—the community and the nation. The World Health Organization, on a global level, and national organizations like AARP in the United States have developed guides to age-friendly homes, communities, and societies. Many of their assessments and recommendations are relevant and applicable cross culturally. New regulations for construction of home and business environments, subsidization of age-friendly renovations, and macrolevel initiatives to promote physical activity, fight obesity, and manage chronic health conditions can be implemented on community and national levels and adapted for cultural acceptability as necessary.

## 5. Conclusions

Globally and nationally, populations are aging. Rising life expectancy is due mainly to advances in medical science and technology (especially in reducing infant and child mortality). The rapid increase in the percent of older adults is due also to decades-long declines in fertility. The world and nations are aging, quickly and permanently, and this must be planned for.

Falling has significant and negative consequences for older adults and their families, communities, and societies. These consequences affect families, communities, and societies along several dimensions, including economic, psychosocial, and health care. Age is strongly related to falling; as populations age, it is more important than ever to take evidence-based steps to change knowledge, attitudes, and behaviors to reduce fall risk at home, in the community, and in society.

Data in this study came from a nonrepresentative sample of older Thai adults in one province of southern Thailand. Thus, generalizability is limited. Replication in the three other regions of Thailand would help assess both validity and reliability; more representative samples would also be advantageous.

Some results of this study of community-dwelling older Thais support prior research in other nations and thus add to the knowledge base of falls, fall risk, and fall-reduction strategies that have cross-cultural applicability. At the same time, some results vary from prior research and show the necessity of cultural awareness. It is only through such awareness that some questions—for example, why are the least- and most-educated Thai elderly more likely to fall?—will find answers, and only through such awareness can evidence-based interventions be developed to adapt to ensure cultural acceptability.

## Figures and Tables

**Table 1:** Demographic characteristics of the sample.

Variable	Number (*n*=406)	Percent
Age		
60–64	194	47.8
65–69	212	52.2
Mean: 64.6 years		
Standard deviation: 3.2 years		

Gender		
Male	175	43.1
Female	231	56.9

Residential location		
City	147	36.2
Town	259	63.8

Marital status		
Single	49	12.1
Couple	126	31.0
Married	190	46.8
Divorced	14	3.4
Separated	27	6.7

Education		
Uneducated	24	5.9
Primary school	195	48.0
Some secondary school	71	17.5
High school graduate	43	10.6
Vocational education	22	5.4
Bachelor's degree	6	1.5
Master's degree	45	11.1

Occupational status		
Unemployed	143	35.2
Pensioner	36	8.9
Merchant	62	15.3
Employee	51	12.6
Gardener	114	28.1

Residence		
Own house	327	80.5
Relative's house	50	12.3
Rental house	29	7.1

Number of family members in household		
Range: 1–9		
Mean: 3.51		
Standard deviation: 1.53		

Living arrangement		
Alone	9	1.2
Spouse	268	35.1
Children	265	34.9
Nephew/niece	168	22.0
Cousin	26	3.4
Others	26	3.4

Income (Thai baht per month, 1000 baht = US$ 30–31)		
Minimum: 500		
Maximum: 112,000		
Mean: 10,953		
Standard deviation: 12,520		

Main source of income		
Children	181	25.4
Spouse	68	9.5
Cousin	17	2.4
Pension	38	5.3
Government support	219	30.7
Working	190	26.6

Health service provider		
Government hospital	405	99.8
Private hospital	1	0.2

**Table 2:** Health status of the sample.

Variable	Number (*n*=406)	Percentage
Body mass index		
Underweight (<17.50 kg/m^2^)	3	0.7
Normal weight (17.50–22.99 kg/m^2^)	146	36.0
Overweight (23.00–27.99 kg/m^2^)	192	47.3
Obese (≥28 kg/m^2^)	65	16.0
Minimum: 15.56 kg/m^2^		
Maximum: 43.75 kg/m^2^		
Mean: 24.60 kg/m^2^		
Standard deviation: 4.15 kg/m^2^		

Vision		
Normal	249	61.3
Abnormal	157	38.7
Presbyopia	98	51.9
Blurry vision	64	33.9
Cataract	12	6.3
Glaucoma	3	1.6
Pterygium	12	6.3

Hearing		
Normal	367	90.4
Abnormal	39	9.6
Hearing aids	6	17.1
Deaf	29	82.9

Diagnosis of chronic condition		
No	162	39.9
Yes	244	60.1
Diabetes	76	19.2
Heart	18	4.6
Hypertension	167	42.3
Hyperlipidemia	99	25.1
Benign prostate hypertrophy	35	8.9

Medication use		
No	138	34.0
Yes	268	66.0
Antianalgesic drugs	51	11.0
Bone and joint medicine	15	3.21
Antidiuretic drugs	6	1.3
Antihyperglycemic drugs	77	16.6
Cardiac medications	16	3.4
Antihypertensive drugs	168	36.2
Antihyperlipidemia drugs	96	20.7
Antidepressive	1	0.2
Sleeping pill	14	3.0
Others	20	4.3

Alcohol drinking		
No	374	92.1
Yes	32	7.9

**Table 3:** Falls history.

Event	Number (*n*=406)	Percentage
Falls since 60 years old		
None	300	73.9
Some	106	26.1
1 time	57	14.0
2 times	29	7.1
3 times	11	2.7
4 times	5	1.2
5 times	2	0.5
6 times	1	0.2
10 times	1	0.2

Falls in previous 6 months		
None	360	88.7
Some	46	11.3
1 time	33	8.1
2 times	9	2.2
3 times	3	0.7
4 times	1	0.2

Falls in previous 3 months		
None	376	92.6
Some	30	7.4
1 time	22	5.4
2 times	7	1.7
3 times	1	0.2

Attributed general causes of falls		
Muscle weakness		
No	339	83.5
Yes	67	16.5
Medication side effect		
No	334	82.3
Yes	72	17.7
Medical condition/lack of assistive devices		
No	357	87.9
Yes	49	12.1
Hazardous home environment		
No	309	76.1
Yes	97	23.9

Specific causes of falls		
Slippery shoes	51	10.8
Contact with other person/object	47	10.0
Slippery floor	40	8.5
Fatigue	39	8.3
Headache	25	5.3
Blurry vision	24	5.1
Muscle pain	24	5.1
Muscle weakness	22	4.7
Presbyopia	20	4.2
Dizziness	17	3.6
Slope/uneven ground	15	3.2
Cramp	13	2.8
Numb leg	13	2.8
Hard heeled shoes	13	2.8
Flushing toilet	12	2.5
Slippery bathroom	11	2.3
Foot abnormality	10	2.1
Long dress or sarong	10	2.1
Stumbling	9	1.9
Cesspool	9	1.9
Drowsiness	8	1.7
In a hurry to void	8	1.7
Foot numbness	7	1.5
General numbness	6	1.3
Poor lighting	5	1.1
Stairs	5	1.1
Muscle tension	3	0.6
High-heeled shoes	3	0.6
Skewness	2	0.4

Falls location		
Outdoor	69	65.1
Indoor	25	23.6
Both	12	11.3

Indoor falls location		
Kitchen	22	30.6
Bedroom	14	19.4
Living room	11	15.3
Terraces	11	15.3
Bathroom	7	9.7
Stairs	6	8.3
Roof	1	1.4

Outdoor falls location		
House surroundings	52	69.3
Footpath/sidewalk	12	16.0
Street/road	4	5.3
Hospital/clinic	2	2.7
Sky walk/overpass	2	2.7
Mall/supermarket	1	1.3
Theatre	1	1.3
Temple	1	1.3

Falls-related health problems		
None	3	2.8
Some	103	97.2
Physical consequences		
Swelling/bruising	63	50.0
Pain	30	23.8
Abrasion	15	11.9
Fracture/dislocation	10	7.9
Laceration	6	4.8
Head injury	2	1.6

Treatment		
Buying drugs at pharmacy	29	27.4
Hospital	28	26.4
No treatment	26	24.5
Resting at home for 1-2 days	14	13.2
Resting for “a while”	6	5.7
Clinic	3	2.8

**Table 4:** Likelihood of falling by selected factors.

Variable	Falling, number (%)		*p* value
	Never	Ever	
Residential location			<0.001
City	88 (59.9)	59 (40.1)	
Town	212 (81.9)	47 (18.1)	

Gender			0.19
Male	135 (77.1)	40 (22.9)	
Female	165 (71.4)	66 (28.6)	

Age			0.029
60–64	153 (78.9)	41 (21.1)	
65–69	147 (69.3)	65 (30.7)	

Marital status			0.9
Single	34 (69.4)	15 (30.6)	
Couple	97 (77.0)	29 (23.0)	
Married	138 (72.6)	52 (27.4)	
Divorced	10 (71.4)	4 (28.6)	
Separated	21 (77.8)	6 (22.2)	

Education			0.009
Uneducated	19 (79.2)	5 (20.8)	
Primary school	137 (70.3)	58 (29.7)	
Secondary school	60 (84.5)	11 (15.5)	
High school graduate	38 (88.4)	5 (11.6)	
Vocational education	12 (54.5)	10 (16.7)	
Bachelor's degree	5 (83.3)	1 (16.7)	
Master's degree	29 (64.4)	16 (35.6)	

Residence			0.45
Own house	241 (73.7)	86 (26.3)	
Relative's house	35 (70.0)	15 (30.0)	
Rental house	24 (82.8)	5 (17.2)	

Number of family members in household			0.13
1 person	9 (52.9)	8 (47.1)	
2–4 persons	228 (75.0)	76 (25.0)	
5+ persons	63 (74.1)	22 (25.9)	

Living arrangement			0.08
Spouse	203 (75.7)	65 (24.3)	
Children	199 (74.8)	67 (25.2)	
Nephew/niece	118 (70.2)	50 (29.8)	
Cousin	16 (61.5)	10 (38.5)	
Others	20 (76.9)	6 (23.1)	
Alone	5 (55.6)	4 (44.4)	

Occupation			0.59
Unemployed	99 (69.2)	44 (30.8)	
Pensioner	27 (75.0)	9 (25.0)	
Merchant	48 (77.4)	14 (22.6)	
Employee	41 (80.4)	10 (19.6)	
Gardener	85 (74.6)	29 (25.4)	

Income			0.5
Below poverty line (<2,647 baht/month)	33 (70.2)	14 (29.8)	
Above poverty line (2,647+ baht/month)	267 (74.4)	92 (25.6)	

BMI			0.046
Underweight (<17.50 kg/m^2^)	3 (100)	0	
Normal weight (17.50–22.99 kg/m^2^)	115 (78.8)	31 (21.2)	
Overweight (23.00–27.99 kg/m^2^)	142 (74.0)	50 (26.0)	
Obese (≥28 kg/m^2^)	40 (61.5)	25 (38.5)	

Vision			0.001
Normal	198 (79.5)	51 (20.5)	
Abnormal	102 (65.0)	55 (35.0)	
Presbyopia	63 (64.3)	35 (35.7)	
Blurry vision	41 (64.1)	23 (35.9)	
Cataract	7 (58.3)	5 (41.7)	
Glaucoma	3 (100.0)	0	
Pterygium	7 (58.3)	5 (41.7)	
Hearing			0.065
Normal	276 (75.2)	91 (24.8)	
Abnormal	24 (61.5)	15 (38.5)	
Hearing aids	5 (83.3)	1 (16.7)	
Deaf	17 (58.6)	12 (41.4)	

Diagnosis of chronic condition			<0.001
No	144 (88.9)	18 (11.1)	
Yes	156 (63.9)	88 (36.1)	
Diabetes	53 (69.7)	23 (30.3)	
Heart	12 (66.7)	6 (33.3)	
Hypertension	105 (62.9)	62 (37.1)	
Hyperlipidemia	64 (64.6)	35 (35.4)	
Benign prostate hypertrophy	21 (60.0)	14 (40.0)	

Medication use			<0.001
No	125 (90.6)	13 (9.4)	
Yes	175 (65.3)	93 (34.7)	
Antianalgesic drugs	29 (56.9)	22 (43.1)	
Bone and joint medicine	5 (33.3)	10 (66.7)	
Antidiuretic drugs	1 (16.7)	5 (83.3)	
Antihyperglycemic drugs	53 (68.8)	24 (31.2)	
Cardiac medications	13 (81.3)	3 (18.8)	
Antihypertensive drugs	108 (64.3)	60 (35.7)	
Antihyperlipidemia drugs	62 (64.3)	34 (35.4)	
Antidepressive	1 (100.0)	0	
Sleeping pill	11 (78.6)	3 (21.4)	
Others	12 (60.0)	8 (40.0)	

Alcohol drinking			0.051
No	281 (75.1)	93 (24.9)	
Yes	19 (59.4)	13 (40.6)	

Muscle weakness			<0.001
No	300 (88.5)	39 (11.5)	
Yes	0	67 (100.0)	
Weak		22 (33.8)	
Fatigue		39 (60.0)	
Cramp		13 (20.0)	
Numb leg		13 (20.0)	
Numb foot		7 (10.8)	
Muscle tension		3 (4.6)	

Medication side effect			<0.001
No	319 (95.5)	15 (4.5)	
Yes	42 (58.3)	30 (41.7)	
Headaches		25 (34.2)	
Blurry vision		24 (32.9)	
Drowsiness		8 (11.0)	
Diuretic		8 (11.0)	
Muscle aches		24 (32.9)	
Numbness		6 (8.2)	
Dizziness		17 (23.3)	

Medical condition/lack of assistive devices			<0.001
No	300 (84.0)	57 (16.0)	
Yes	0	49 (100.0)	
Myopia		18 (36.7)	
Presbyopia		20 (40.8)	
Skewness		2 (4.1)	
Abnormal foot		10 (20.4)	

Home hazard environment			<0.001
No	300 (97.1)	9 (2.9)	
Yes	0	97 (100.0)	
Slippery floor		40 (41.2)	
Slope/uneven ground		15 (15.5)	
Stumbling		9 (9.3)	
Contact with other person/object		47 (48.5)	
Poor lighting		5 (5.2)	
Stairs		5 (5.2)	
Flushing toilet		12 (12.4)	
Cesspool		9 (9.3)	
Slippery bathroom		11 (11.3)	

Footwear/clothing			<.0001
No	300 (89.6)	35 (10.4)	
Yes	0	71 (100.0)	
Slippery shoes		51 (71.8)	
High-heeled shoes		3 (4.2)	
Hard-heeled shoes		13 (18.3)	
Long dress/sarong		10 (14.1)	

Indoor area			<0.001
No	300 (85.2)	52 (14.8)	
Yes	0	54 (100.0)	
Bedroom		14 (25.5)	
Kitchen		22 (40.0)	
Living room		11 (20.0)	
Terraces		11 (20.0)	
Stairs		6 (10.9)	
Bathroom		7 (12.7)	
Roof		1 (1.8)	

Outdoor area			<0.001
No	300 (86.7)	46 (13.3)	
Yes	0	60 (100.0)	
Home surrounding		52 (82.5)	
Foot path		12 (19.0)	
Department store		1 (1.6)	
Sky walk/overpass		2 (3.2)	
Street/road		4 (6.3)	
Theatre		1 (1.6)	
Temple		1 (1.6)	
Hospital/clinic		2 (3.3)	

## Data Availability

Data availability inquiries should be addressed to the first author.
